# Themes in biomedical natural language processing: BioNLP08

**DOI:** 10.1186/1471-2105-9-S11-S1

**Published:** 2008-11-19

**Authors:** Dina Demner-Fushman, Sophia Ananiadou, K Bretonnel Cohen, John Pestian, Jun'ichi Tsujii, Bonnie Webber

**Affiliations:** 1 US National Library of Medicine, 8600 Rockville Pike, Bethesda, MD 20894, USA; 2 University of Manchester and National Centre for Text Mining, 131 Princess Street, M7 1DN, UK; 3 University of Colorado Health Sciences Center, US mail: PO Box 6511, Mail Stop 8303, Colorado, USA; 4 Computational Medicine Center, Cincinnati Children's Hospital and Medical Center, 3333 Burnet Avenue, Cincinnati, Ohio 45229-3039, USA; 5 University of Tokyo, Japan and University of Manchester, 131 Princess Street, M7 1DN, UK; 6 University of Edinburgh, 2 Buccleuch Place, Edinburgh EH8 9LW, UK

## Background

A recent posting to the BioNLP mailing list notes that the past few months of 2008 have seen the appearance of over fifty papers on biomedical natural language processing/text mining (BioNLP). This number (which included medical, as well as genomic work) represents about as many papers on genomic language processing as existed in all of PubMed at the end of 2003 [1] – just five years ago, and the current supplement in *BMC Bioinformatics *presents another ten! These papers have in common the fact that they are follow-on work to papers originally published in the proceedings of the BioNLP 2008 workshop at the annual meeting of the Association for Computational Linguistics (ACL). All have gone through a separate rigorous review process and represent an advance beyond the work originally presented at the workshop. Like the annual BioNLP workshop itself, they represent a wide cross-section of the type of work that goes on in BioNLP today.

Annual BioNLP workshops have been held since 2002 in conjunction with the annual meeting of the ACL or its North American chapter. Whereas other venues, such as NLP sessions at biomedical informatics and computational biology meetings, provide excellent opportunities for presenting applications of NLP in the biomedical domain, the BioNLP workshop has consistently been a venue for presenting work in areas of fundamental BioNLP that is innovative and challenging from an NLP perspective.

Research in computational linguistics in the biomedical domain traditionally focuses on two major areas: fundamental advances in language processing; and application of language processing methods to bridge the gap between basic biomedical research, clinical research, and translation of both types of research into practice. The expanded and updated versions of the best papers in both areas presented at the BioNLP 2008 workshop have been selected by the Program Committee for publication in this supplement to *BMC Bioinformatics*. Of 19 full papers and 5 posters submitted to the workshop, 10 were accepted as full papers and 18 as poster presentations. The combined expertise of the program committee allowed for providing three thorough reviews for each paper. The exceptionally high quality manuscripts accepted for presentation covered a wide area of subjects in clinical and biological areas, as well as methodological issues applicable to both sublanguages. Separately, those authors were invited to submit papers describing significant advances beyond their original papers and posters for inclusion in this supplement.

In addition to the presented papers and posters, BioNLP 2008 featured two keynote talks.

John Hutton, MD, Professor of Pediatrics and Director of Biomedical Informatics, Cincinnati Children's Hospital, presented a large academic medical center perspective on computational linguistics approaches to enhancing Clinical Decision Support Systems.

LTC Hon Pak, MD, Chief, Advanced Information Technology Group Telemedicine and Advanced Technology Research Center (TATRC), U.S. Army Medical and Material Research Command, discussed the need for NLP in Department of Defense Military Health System (MHS).

## Summary of the selected contributions to the supplement

Two papers are dedicated to relation extraction. Airola *et al. *[2] present a new graph-kernel approach to protein-protein interaction extraction, whereas Roberts *et al. *[3] treat clinical relationship extraction as a classification task, training classifiers to assign a relationship type to an entity pair assuming perfect entity recognition, as given by the entities in the manually annotated reference standard.

As automatic named entity recognition (NER) significantly impacts all subsequent processes in BioNLP pipelines, it continues to be an active area of research. Corbett and Copestake [4] achieve ~60% recall at 95% precision, and ~60% precision at 90% recall in identifying chemical named entities using cascaded classifiers. Sasaki *et al. *[5] combine dictionary-based and statistical methods for recognition of protein names.

Many NLP applications are believed to require determination of the sense of a recognized entity. For example, an automatic system that links research evidence to a patient's record needs information on the sense of discharge (Discharge [Body Substance] or Patient Discharge [Health Care Activity]) in a given context to provide appropriate evidence. Wang and Matthews [6] demonstrate benefits of species disambiguation for recognition of protein names. Stevenson *et al. *[7] adapt an open-domain word sense disambiguation (WSD) system to the biomedical domain by augmenting it with additional domain-specific and domain-independent information sources.

Creation of domain-specific resources is an important task that stimulates BioNLP research, once the resources are made available to the community. To that end, Tsuruoka *et al. *[8] present an active learning framework for acceleration of the annotation process, and Vincze *et al. *[9] describe their work on creation of a corpus annotated for negation and uncertainty.

Determining authors' confidence in their findings (certainty of the conclusion statement, otherwise approached as recognition of speculative language, or hedging) is another important area of research. Kilicoglu and Bergler [10] use lexico-syntactic patterns and semi-automatic weighting of hedging cues to recognize speculative language. Finally, application of NLP methods to classic information retrieval problems such as automatic indexing of biomedical literature is presented by Neveol *et al. *[11].

## Competing interests

The authors declare that they have no competing interests.
